# Scanning structural mapping at the Life Science X-ray Scattering Beamline

**DOI:** 10.1107/S1600577521013266

**Published:** 2022-01-17

**Authors:** Lin Yang, Jiliang Liu, Shirish Chodankar, Stephen Antonelli, Jonathan DiFabio

**Affiliations:** a National Synchrotron Light Source II, Brookhaven National Laboratory, 745 Brookhaven Avenue, Upton, NY 11973, USA

**Keywords:** structural mapping, imaging, SAXS/WAXS

## Abstract

This work describes the instrumentation and software for microbeam scattering and structural mapping at the Life Science X-ray Scattering (LiX) beamline at NSLS-II. Using a two-stage focusing scheme, an adjustable beam size between a few micrometres and a fraction of a millimetre is produced at the sample position. Scattering data at small and wide angles are collected simultaneously on multiple Pilatus detectors. A recent addition of an in-vacuum Pilatus 900k detector, with the detector modules arranged in a C-shaped configuration, has improved the azimuthal angle coverage in the wide-angle data. As an option, fluorescence data can be collected simultaneously. Fly scans have been implemented to minimize the time interval between scattering patterns and to avoid unnecessary radiation damage to the sample. For weakly scattering samples, an in-vacuum sample environment has been developed here to minimize background scattering. Data processing for these measurements is highly sample-specific. To establish a generalized data process workflow, first the data are reduced to reciprocal coordinates at the time of data collection. The users can then quantify features of their choosing from these intermediate data and construct structural maps. As examples, results from in-vacuum mapping of onion epidermal cell walls and 2D tomographic sectioning of an intact poplar stem are presented.

## Abstract 2

Instrumentation and software for supporting microbeam structural mapping at the LiX beamline are presented.

## 1. Introduction

Raster scanning is a common method for characterizing the spatial distribution of components in heterogenous samples. Scanning imaging is in wide use at synchrotron beamlines, based on a variety of contrast mechanisms (*e.g.* fluorescence emission, absorption, phase contrast) on length scales from nanometres to sub-millimetre. At the Life Science X-ray Scattering (LiX) beamline, we focus on scanning imaging of biological tissues using scattering contrast. Biological tissues are hierarchical structures, in which many components exhibit periodicity and therefore produce diffraction peaks. For instance, collagen and minerals in bones produce peaks in the scattering data at small and wide angles, respectively (*e.g.* Paris, 2008 ▸). Another example is cellulose, which is abundant in plant cell walls, the scattering data from which contain information about the crystalline cellulose structure at the molecular scale at wide scattering angles, as well as the organization of the cellulose fibrils at small angles (*e.g.* Rongpipi *et al.*, 2019 ▸). This information on material abundance and distribution can then be used to help understand how external factors influence the structure. For instance, in bio­energy research, it is of great interest to understand how cell wall structures in the feedstock can be affected by mechanical processing and chemical treatments (Cheng *et al.*, 2015 ▸), as well as genetic modification (Cai *et al.*, 2016 ▸).

Although synchrotron-based imaging and microscopy have made impressive progress in recent years (*e.g.* Hémonnot & Köster, 2017 ▸), scattering methods may still have the edge in near-micrometre-resolution characterization of biological tissues. Due to the structural diversity and complexity in these samples, making sense of high-resolution images often requires advanced image analysis (Polo *et al.*, 2020 ▸). In contrast, scattering measurements provide averaged structural characterization within the beam spot, yet can still be performed with moderate spatial resolution. Scanning imaging based on scattering contrast therefore presents an opportunity to correlate the morphological information at near-micrometre resolution in real space with the structural information on molecular scales from reciprocal space.

Scattering-based scanning imaging has been discussed in the literature ever since it became feasible to achieve small spot sizes for diffraction measurements. The idea of using small beams for histology was explored well before the advent of synchrotron sources (Eckling & Kratky, 1930 ▸). But of course practical use of this method only became possible after small beam sizes could be routinely achieved at modern synchrotron beamlines (*e.g.* Riekel, 2000 ▸). In recent years, tomographic reconstruction based on both isotropic (Schroer *et al.*, 2006 ▸; Jensen *et al.*, 2011 ▸) and anisotropic (Liebi *et al.*, 2015 ▸; Schaff *et al.*, 2015 ▸) scattering have also been reported. However, the application of scattering-based structural mapping seems to have flourished only in specialized fields, such as the studies of bone and wood. This is at least partly limited by the sample-specific data analysis, which is not universally applicable for all types of structures and can be challenging for non-expert beamline users. The availability of appropriate instrumentation may be another limiting factor. Some studies (Liu *et al.*, 2016 ▸) have been performed using instruments dedicated to macro-molecular crystallography, with limited data coverage at low-*q*.

The LiX beamline aims to meet the instrumentation need for optimized scattering-based scanning imaging, with emphasis on inclusion of high-quality scattering data at both small and wide scattering angles, as well as the software need for a generic workflow for data processing and analysis. As reported previously (DiFabio *et al.*, 2016 ▸), this instrument is designed to support multiple types of measurements on biological samples. The instrumentation and software for biomolecular solution scattering have been detailed elsewhere (Yang *et al.*, 2020 ▸). Here, we describe the instrumentation and software we have developed to support structural mapping of biological tissues. In particular, we will highlight our efforts in extending the range of scattering vectors (*q*) and minimizing the scattering background to enable measurements on weakly scattering tissue samples.

## 2. Instrumentation and control

At the LiX beamline the X-ray beam is first focused by a pair of mirrors in the Kirkpatrick–Baez configuration to a secondary source aperture (SSA). The beryllium compound refractive lenses (CRLs) then image the SSA to the sample position (DiFabio *et al.*, 2016 ▸). An on-axis sample viewer is installed to monitor the sample and for low-resolution beam characterization. Scattering data at both small and wide scattering angles are collected simultaneously, optionally together with X-ray fluorescence data.

### 2.1. CRL transfocator

**Figure 1:**
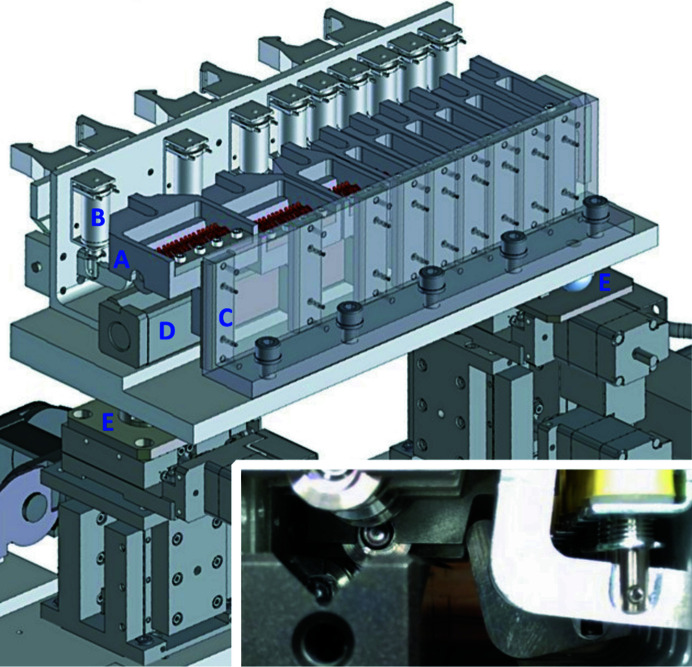
CRL transfocator at LiX. The lenses are organized into nine lens holders. Each holder (A) can be moved into or out of the beam using a bis­table solenoid (B) and guided by a linear rail (C). In the ‘in’ position, the lenses are pushed by the finger spring (copper-colored) installed in the holder against, and therefore aligned by, a Vee block (D). The transfocator is supported on either end by a stack of *x*–*y* stages via kinematic mounts (E). The inset is a photograph of the components in the transfocator.

CRLs are now widely used for X-ray focusing. In order to account for the energy dependence of the CRL focal length, a transfocator is typically used to vary the number of lenses in the beam. Several transfocator designs have been described in the literature (Rossat *et al.*, 2010 ▸; Zozulya *et al.*, 2012 ▸; Vaughan *et al.*, 2011 ▸). Some devices are also commercially available. We have adopted an in-vacuum design (Fig. 1 ▸) using bis­table solenoids (Ledex C5-L-272-B-1) to move lens groups into and out of the beam. The solenoids only need to be powered on when the state (in/out) of the lens group needs to be changed, thus avoiding the problem of solenoid heating in vacuum. The lenses are aligned using a Vee block, following the design by Zozulya *et al.* (2012 ▸). The transfocator consists of 43 lenses (RX Optics) of 0.1 mm radius, divided into groups of 1, 2, 4, 8, 16 and 12 lenses, with four additional lenses of radius 0.5 mm to provide finer control on the combined focal length. The solenoids are driven by motor driver boards (Pololu DRV8800) that take inputs from a BeagleBone single-board computer, on which a soft IOC is run to provide *EPICS* (*Experimental Physics and Industrial Control System*) control over the state of the lens groups.

The transfocator is mounted on a long translation stage in a vacuum chamber, so that the distance between the sample and the transfocator can be adjusted in the range of ∼0.5–1.5 m. The total distance between the SSA and the sample is 8 m. Therefore the demagnification by the CRLs can vary from ∼4:1 to 15:1, which determines the required focal length and the corresponding size of the focused beam. This in turn requires an energy-dependent lens configuration. The calculated focal length as a function of the number of lenses and the X-ray energy in the beamline operating range 7–18 keV is shown in Fig. S1 of the supporting information. Since the transfocator cannot provide continuously adjustable focal lengths, the location of the transfocator can be varied to achieve the best focus when necessary, at the expense of slightly altering the demagnification. For microbeam mapping we typically use a beam size of 5 µm, with the SSA partially closed to shape the beam. The divergence-limiting aperture, located just upstream of the CRLs, is also partially closed to limit the beam within the effective acceptance aperture of the lenses, as well as to reduce the beam divergence. A set of guard slits are installed before the on-axis viewer (described below) to ensure a clean background in the small-angle scattering data.

The transfocator needs to be aligned to the incident X-ray beam for optimal performance. This is accomplished using the *x*–*y* stacks of translation stages supporting each end of the transfocator (Fig. 1 ▸). Minor misalignment primarily affects the position of the focused beam. More severe misalignments reduce focused beam intensity when the effective aperture is no longer fully illuminated, which can be used as the basis of transfocator alignment. For alignment we insert the lens groups at either end of the transfocator into the beam to represent its optical axis. We scan the upstream and downstream motors in the same direction, then opposite directions, and consider the position with maximum X-ray transmission as the optimal position. Multiple iterations may be necessary to achieve optimal alignment. Once the transfocator is aligned, the on-axis viewer and a scintilator are used to visualize the beam and help optimize the beam focus. For a more precise measurement of the beam size, a knife-edge scan can be performed.

### 2.2. On-axis sample viewer

**Figure 2:**
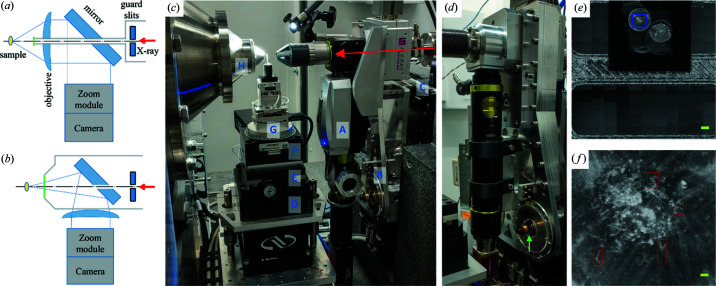
Sample positioning stages and on-axis cameras used for scanning measurements. (*a*) and (*b*) depict the layout of the components in the on-axis viewers shown in (*c*) and (*d*), respectively. The red arrows represent the incident beam. The blue lines show the path of visible light. The gray lines represent the vacuum enclosure and the green line near the sample represents the mica window. The components for the scanning stack are show in (*c*): (A) on-axis viewer, (B) tilt platform, (C) stages for viewer translation, (D) vertical scanning stage, (E) horizontal scanning stage, (D) rotary stage for tomographic data collection, (E) motorized goniometer head and (H) WAXS nose cone. One of the screws for the tilt platform is indicated by the green arrow in (*d*). (*e*) Tiled, full-field sample view assembled from individual on-axis images such as the one shown in (*f*), which corresponds to the blue box in (*e*). The scale bars are 1 mm and 0.1 mm for (*e*) and (*f*), respectively. The user-defined ROIs are shown as red boxes in (*f*).

Direct visualization of the sample is an important aspect of the instrumentation that supports scanning measurements. It allows the experimenter to select regions of interest (ROIs) for measurements and follow the data collection progress. We devised a common support that can mount two different sample viewers (Fig. 2 ▸). In either case, we extended the vacuum beam path as close to the sample as possible to reduce air scattering background (more discussion on this later).

The first viewer [Figs. 2 ▸(*a*) and 2 ▸(*c*)] is based on a QiOptic Optem microscope with a drilled objective and a drilled 45° mirror, so that the X-ray beam can pass through both of these components. A molybdenum tube (1.5 mm in diameter) lines the inside of the apertures in the lens and the mirror to protect the glass components and at the same time serves as a vacuum beam path. The tube is terminated on the sample side with a glued-on mica window. The working distance of the objective is 34 mm. The molybdenum tube sticks out of the objective, protected by a 3D-printed hood to further reduce the in-air X-ray path length.

The second viewer [Figs. 2 ▸(*b*) and 2 ▸(*d*)] utilizes a drilled mirror only. It is based on a different QiOptiq Optem microscope, which is located on the opposite side of the mirror from the sample. The vacuum path is again extended close to the sample and terminated with a mica window. The beam tube exit is much larger in this case, encompassing the light-gathering cone of the objective. The magnification of this viewer can be adjusted using the zoom module, as well as by replacing the objective, which also changes the working distance of the viewer.

Both viewers are interchangeably mounted on a support stage that adjusts the position of the microscope with respect to the beam, as well as its orientation. The tilt motions are realized using two screws [Fig. 2 ▸(*d*)] that move the two corners of a triangular support for the viewer. Pseudo motors are implemented in the *Bluesky* software (Arkilic *et al.*, 2017 ▸) layer to translate the linear motion of the screws into the tilt motions of the microscope, corresponding to the two screws moving in the same direction or in opposite directions.

These viewers inevitably have limited fields of view that are typically much smaller than the sample size, even at the lowest magnification. To facilitate the selection of ROIs for automated measurements, we tile a series of on-axis images into a full-field view [Fig. 2 ▸(*e*)] of the sample. These images are collected in a raster scan using the camera as the detector. Individual images are pasted into a large blank image based on the motor position recorded in the scan. In order to position these images correctly, a calibration measurement is performed first, by imaging the corner of a rectangular aperture, moving by a known distance along the direction of rows/columns in the image. By using image processing, the position of the edge can be found automatically in the ‘before’ and ‘after’ images to determine the conversion between the actual distance and the observed motion in the on-axis image. The user can then select ROIs for measurements by specifying the pixel positions in the tiled image. Once another image is taken using a scintillator to determine the beam position in the on-axis view, beamline software can translate the pixel positions of these ROIs back to actual motor positions and carry out the measurements [Fig 2 ▸(*f*)].

### 2.3. Scanning stack

The scanning stack [Fig. 2 ▸(*c*)] is designed to support multiple sample formats. It is installed on a base shared with the sample handler for solution scattering (Yang *et al.*, 2020 ▸), providing coarse *x* (horizontal and perpendicular to the beam) and *z* (along the beam direction) motions. The *z*-stage helps to adjust the sample position so that the image seen by the on-axis viewer is in focus, which also ensures a constant sample-to-detector distance. Three Newport stages provide the motions required for scanning and tomography measurements: *x* (VP-25XA), *y* (vertical, GTS30V) and *R*_*y*_ (rotation about *y* axis, URS75BCC). A motorized goniometer head consisting of four SmarAct stages that provide translation (*x* and *z*) and tilt motions (*T*_*x*_ and *T*_*z*_) keep the sample aligned to and centered on the *R*_*y*_ axis. Doing so helps to minimize the variation in the scan range in tomography data collection, where the sample needs to be continuously rotated about the *R*_*y*_ axis (vertical). In 2D scanning of flat samples, *T*_*x*_ and *R*_*y*_ are used to keep the sample plane perpendicular to the beam, so that the sample is always at a constant *z* position during scanning. The stack can be removed from the *R*_*y*_ stage and above, so that other multi-position sample holders can be installed instead.

We use standard 3 mm and 5 mm light-emitting diodes (LEDs) to provide sample illumination in 3D-printed holders that are mounted in close proximity to the sample. A magnetic sample mount is used at the top of the goniometer head to interface with various sample formats. As a future upgrade, the six-axis Staubli robot (Yang *et al.*, 2020 ▸) used for solution scattering will be used to change samples in remote measurements.

Fly scanning is preferred when the time required for stage motion becomes significant compared with the measurement time per data point. In scanning measurements on tissue samples at LiX, the exposure time for scattering data collection is typically a fraction of a second. We have therefore implemented fly scanning using the PVT (a given series of position, velocity and time interval) trajectory functionality of the Newport XPS controller. Each 2D scan is broken into single-motor linear PVT trajectories with adjoining motor motions to transition into the next line in the scan. The detectors are triggered as the fast-axis motor reaches each point during the execution of the trajectory. Data are written to HDF5 (HDF Group, 2018 ▸) format using the HDF plugin of the *EPICS areaDetector* plugin (Rivers 2010 ▸), and later packaged into larger HDF5 files that contain data from all detectors. The software for fly scanning and data packing can be found on the LiX github site (https://github.com/NSLS-II-LIX) under profile_collection.

### 2.4. In-vacuum scanning

**Figure 3:**
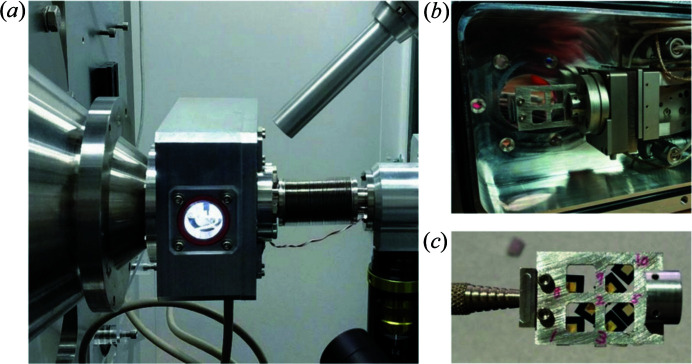
In-vacuum scanning measurements are performed in a small vacuum chamber mounted directly on the WAXS nose cone (*a*), with internal sample illumination and a kapton window that permits fluorescence data collection. The position stages with the magnetically mounted sample holder are shown in (*b*). The sample holder is loaded into the vacuum chamber using a tool with a threaded tip (*c*).

Though the long-term plan at the LiX is to develop a cryo-capable instrument like the OMNY (Holler *et al.*, 2018 ▸), we currently have a simple in-vacuum stack mainly intended for minimizing the scattering background. This is important for measurements on weakly scattering thin tissue sections. In the current implementation, the sample motions are limited to the *x*–*y* translations perpendicular to the beam and the *R*_*x*_ rotation for orienting the sample with respect to the beam. All motions are realized using SmarAct stages (Fig. 3 ▸). The sample is attached to the scanning stack using a magnetic mount. A hand tool with a threaded end is used to mount or remove the sample though the small port (∼1 inch diameter) on the vacuum chamber. Again LEDs are used to provide sample illumination. The upstream LEDs are glued into the chamber lid and point at the sample. The downstream LEDs point at the sample at a much steeper angle, in order to create a more dark-field-like illumination that accentuates contrast.

We are in the process of implementing fly scanning for the in-vacuum measurements. One of the XPS driver cards (DRV00P) can produce step and direction signals, which can then be used as input for the SmarAct SDC2 driver. Therefore we are able to use the same fly scanning code that we developed for in-air scanning.

Sample exchange is time consuming due to the need for breaking then re-establishing the vacuum environment. Nonetheless, the in-vacuum stack can cover a ∼25 mm × 25 mm area, in which multiple samples can be mounted. With a typical beam size of 5 µm, we generally only need to change the samples twice a day. Python functions have been developed to automate the venting and evacuation of the sample chamber, with a built-in safeguard against user error that might lead to accidental venting of the WAXS detector chamber.

### 2.5. Detectors and beam monitors

**Figure 4:**
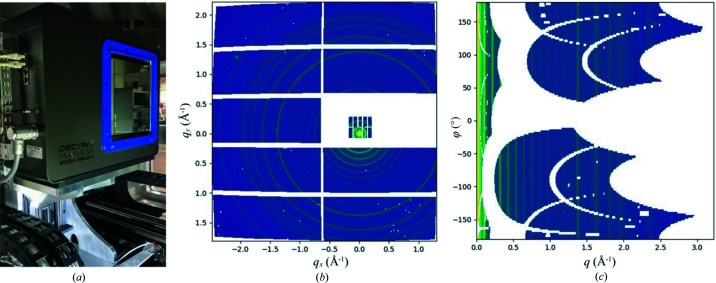
SAXS/WAXS data collection at LiX. (*a*) In-vacuum Pilatus 900k detector, located in the WAXS chamber downstream of the nosecone visible in Fig. 2 ▸(*c*), for WAXS data collection. The SAXS detector is a Pilatus 1M and not shown. (*b*) SAXS/WAXS patterns collected from a silver behenate sample, shown as maps sharing the same reciprocal coordinates (in the plane perpendicular to the incident beam), after scattering intensity has been scaled between the two detectors. (*c*) The same scattering patterns can also be represented by intensity maps using the scattering vector *q* and the azimuthal angle φ as coordinates.

We use multiple Pilatus detectors to maximize the coverage in reciprocal space. The SAXS detector is a Pilatus 1M, located at the end of the bellow-based flight path. Initially, two Pilatus 300k detectors, located on either side of the SAXS detector (DiFabio *et al.*, 2016 ▸), were used for recording wide-angle scattering data. This worked well for isotropic scattering. However, scattering from biological tissues is typically anisotropic. This detector configuration only covers a narrow range of azimuthal angles at wide scattering angles and can therefore potentially miss a lot of useful information, depending on the orientation of the underlying structures in the sample. In order to improve the azimuthal coverage at wide scattering angles, we installed an in-vacuum Pilatus 900k detector [Fig. 4 ▸(*a*)], which is essentially a Pilatus 1M with a module in the center row removed to allow scattered photons at small scattering angles to pass through.

In a typical scanning experiment, the sample-to-detector distance is ∼3 m for SAXS and for ∼0.3 m for WAXS, to provide the desired *q*-range ∼0.006–3 Å^−1^. This large distance difference, compounded by the fact that the clear aperture on the WAXS detector is rectangular whereas the shape of the SAXS detector is close to a square, still leaves gaps in the detector coverage in reciprocal space [Fig. 4 ▸(*b*)]. While one of the original Pilatus 300k detectors can be used (downstream of the 900k) to fill in more gaps, due to the short distance between the sample and the 900k detector and its thickness (∼14 cm along the direction of the incident beam), the exit end of the 900k aperture shadows a much larger area than what is missed by its sensing modules. Ideally, a larger WAXS detector would be employed, with a square clear aperture and located further away from the sample, to minimize the gaps in reciprocal space coverage.

In cases where the WAXS data are expected to be centrosymmetric, the effective coverage of the reciprocal space is extended. In addition, the detector gaps can often be filled by interpolation, based on the assumption that the scattering intensity varies continually. More sophisticated ‘healing’ of scattering patterns is also possible (Liu *et al.*, 2017 ▸). As a routine step of our data processing workflow, we create intensity maps using reciprocal coordinates [*q* versus azimuthal angle φ, Fig. 4 ▸(*c*)] for each set of scattering patterns. These maps provide a common basis for further feature extraction (*e.g.* peak position or intensity in line profiles derived from these maps) to formulate structural maps.

X-ray fluorescence data provide complementary elemental distribution in the sample and can be easily incorporated into our data collection. Specialized fluorescence imaging beamlines typically have a fluorescence detector installed at 90° with respect to the incident beam to minimize scattering background. This is not practical for simultaneous scattering measurements on flat samples, which need to be perpendicular to the beam to minimize the illuminated volume on the sample, and therefore to achieve the best possible spatial resolution. Therefore, we must resort to alternative methods to manage scattering background, by leaving only a small portion of the beam path in air, and to have a detector capable of high count rates. We currently use a Vortex silicon drift detector with Xspress3 readout electronics from the NSLS-II detector pool. The detector is positioned as close to the sample as practical to maximize the detection solid angle (the sensing area is ∼0.5 cm^2^). In the specific example shown in Fig. 3 ▸, the detector views the sample at 45° from the beam and is <10 cm from the sample. A dedicated pixel-array detector, similar to the Maia (Ryan *et al.*, 2013 ▸), is being built by the NSLS-II detector group.

We have two beam monitors for recording the beam intensity upstream and downstream of the sample. The incident beam monitor is a diamond quadrant detector (Sydor), located between the CRL transfocator and the guard slits (DiFabio *et al.*, 2016 ▸). It also serves as a beam position monitor. The transmitted beam monitor is a photodiode (Honeywell SD1420) embedded in the beamstop and measures the emission from a YAG scintillator (Yang *et al.*, 2020 ▸). Both devices are read out using electrometers developed by the NSLS-II detector group.

## 3. Examples

As discussed earlier, data processing is a major bottleneck that limits broader application of scattering-based scanning mapping. Ideally, the users need to be able to have immediate feedback during data collection, and revise the data processing workflow accordingly. The long-term plan is that the NSLS-II *Bluesky* software ecosystem will support on-the-fly data processing. In the interim, we perform data processing once a scan is completed and all the data are packaged into a HDF5 file, as done for solution scattering measurements at the beamline (Yang *et al.*, 2020 ▸). The general workflow is to first use the Python packages *py4xs* and *lixtools* to generate pre-processed data, including an azimuthally averaged and merged 1D intensity profile and *q*–φ intensity maps. This is typically the most time-consuming data processing step and requires the scattering geometry to be defined for each detector. Depending on the sample, beamline staff and/or users can then use generic software to extract features (1D or 2D) from the pre-processed data, which are saved and therefore can be quickly recalled, and assemble structural maps.

### 3.1. In-vacuum measurements on an onion epidermal cell wall

During in-air measurements of weakly scattering samples, the background scattering often changes with the sample position as the sample supports shadow the air scattering originating upstream of the sample. This makes background subtraction difficult, if not impossible. Conducting these measurements with the sample under vacuum makes this problem much more manageable. As an example, we present data collected from onion epidermal cell walls. In recent publications, grazing incident scattering and tender X-ray resonant scattering (Ye *et al.*, 2018 ▸, 2020 ▸) have been employed to enhance the scattering signal from these samples. Here we show that, given lowered scattering background, the same structural information can be obtained in the transmission scattering geometry with position sensitivity.

**Figure 5:**
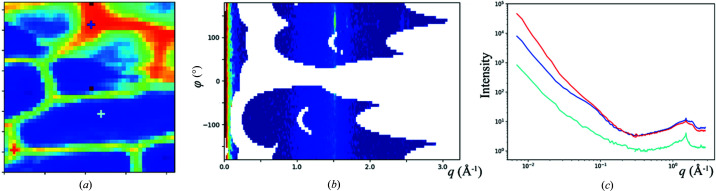
Scattering-based structural mapping of onion epidermal cells. (*a*) Map based on the overall scattering intensity at *q* > 0.1 Å^−1^. Representative scattering data, corresponding to the location labeled by the cyan ‘+’ in (*a*), are shown in (*b*) as a merged *q*–φ intensity map and using a logarithmic color scale. The azimuthally averaged data, from the three locations indicated by ‘+’ signs with the coreesponding color coding in (*a*) are shown in (*c*). The sharp feature at *q* ≃ 1.5 Å^−1^ can be attributed to the naturally occurring wax.

The sample is prepared by suspending the epidermal tissue peeled from an onion over the aperture on the sample support. The onion epidermal cells have primary cell walls, with a thickness on the order of 1 µm (Ye *et al.*, 2018 ▸) and the scattering intensity from which could be easily overwhelmed by air scattering. Even for in-vacuum measurements, the scattering background may still need to be subtracted. This background can come from the sample support (*e.g.* mica or silicon nitride window) and the residual air in the SAXS flight path, where the vacuum pressure is ∼10^−2^ mbar, but it is predictable based on the transmitted beam intensity and therefore can be subtracted easily. The structural map based on overall material abundance (integrated intensity of scattering data at *q* > 0.1 Å^−1^) is shown in Fig. 5 ▸(*a*), with representative scattering data given in Figs. 5 ▸(*b*) and 5 ▸(*c*). Once the scattering background is subtracted, the azimuthally averaged SAXS and WAXS data are combined to span a continuous extended *q*-range from 0.006 Å^−1^ to ∼3 Å^−1^.

The 1D scattering profiles in Fig. 5 ▸(*c*) show basic features similar to those collected from bulk cellulose samples, including the diffraction peaks from crystalline cellulose, as well as the power-law-like intensity decay at low-*q* that spans ≥3 decades in intensity and is characteristic of self-similar multi-scale structures. At the boundaries between cells, the cell walls are oriented along the beam and many more materials contribute to the scattering intensity. In this case a feature is also visible at *q* ≃ 0.1 Å^−1^, consistent with the correlation between cellulose fibrils in primary cell walls observed in scattering measurement on other plants (Kennedy *et al.*, 2007 ▸). However, in our case this peak appears at a lower *q* value, indicating a larger average distance between fibrils. This peak is not visible when the beam is perpendicular to the cell wall, likely due to the lower in-plane number density of fibrils and longer average spacing between them. This is consistent with the observations by Ye *et al.* (2018 ▸), where a Fourier transform of the atomic force microscopy image of the cell wall produced what amounted to the form factor of the cellulose fibril with no inter-fibril correlation. This study also reported a peak at *q* ≃ 0.03 Å^−1^ extracted from resonant scattering data, which was interpreted as cellulose inter-fibril correlation, with enhanced contrast from calcium ions bound to the pectin matrix.

### 3.2. Tomographic cross section of a plant stem

Tomography enables the experimenter to non-destructively explore the internal structure of materials. At synchrotron beamlines, tomography can be implemented with various contrast mechanisms (*e.g.* fluorescence, absorption) and imaging modes (scanning versus full field). Although absorption-based tomography may be the most common, its application to soft matter and biological materials is limited by poor absorption contrast due to the low-*Z* composition of these materials. Scattering-based tomography can help visualize the 3D distribution of periodical structural components in biological tissues, and can be used in tandem with fluorescence tomography that reveals elemental distribution. In general, the orientation of components with structural periodicity introduces additional complications, as the contribution to the scattering signal from each voxel in the sample can vary between projections. Nonetheless, as long as the observed scattering intensity is independent of sample rotation, either because the structure within the voxel is randomly oriented or if the fiber structure has its preferred orientation approximately parallel to the rotation axis, tomographic reconstruction can be solved using generic tomography software packages like *tomopy* (Gürsoy *et al.*, 2014 ▸).

**Figure 6:**
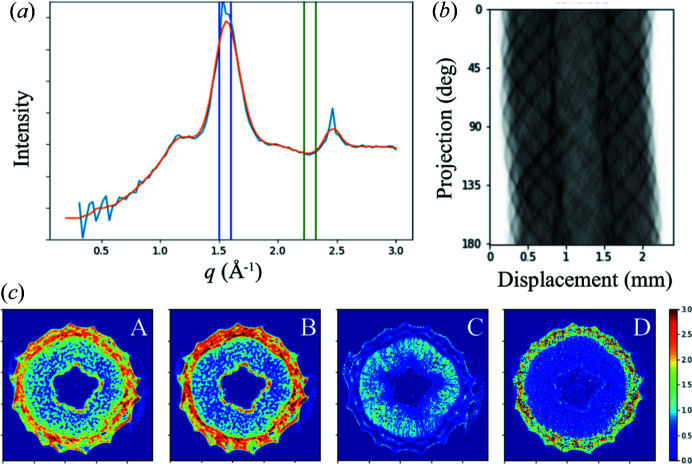
Virtual sectioning of a poplar stem by tomography based on X-ray scattering contrast. (*a*) Assuming the structures in the sample exhibit a fiber geometry, with the fiber axis perpendicular to the incident X-ray beam, we reduce all scattering patterns to 1D intensity profiles (blue line) by azimuthal averaging. These data are further processed by smoothing (orange line) to eliminate sharp features caused by the non-cellulose components in the sample. (*b*) For this experiment, we select the intensity under the cellulose (200) peak at *q* = 1.58 Å^−1^ and the intensity near *q* = 2.27 Å^−1^ to represent the abundance of crystalline cellulose and amorphous components, respectively, as indicated by the blue and green boxes in (*a*). Sinograms are generated based on the estimated material abundance. The crystalline content sinogram is shown as an example. (*c*) Several tomograms are derived from the data using *tomopy*, based on the abundance of (A) crystalline cellulose and (B) amorphous components. (C) is the ratio of (A) over (B), similar to the crystalline index customarily used in cellulose research. For comparison, the tomogram based on absorption contrast is shown in (D).

Here, we present the tomographic sectioning of a poplar stem based on scattering contrast. The sample is mounted on the in-air scanning stack shown in Fig. 2 ▸(*c*). We use the on-axis sample viewer described above to help align the sample to the rotation axis *R*_*y*_, with the growth direction parallel to the rotation axis, therefore perpendicular to the incident beam. This approximately satisfies the requirement of rotation invariance discussed above. We also center the sample on the rotation axis, minimizing the scanning range during sample rotation. The sample is scanned in the *x* direction with a 5 µm step size at every projection angle, which varied from −90 to 90° at 1.5° intervals. For data processing we first performed azimuthal averaging on the scattering patterns for each projection, followed by smoothing to eliminate the sharp diffraction peaks from minerals in the bark [Fig. 6 ▸(*a*)]. Tomographic reconstructions can be performed based on any attributes derived from the scattering intensity, as long as the calculation for the attribute is linear with respect to the contributions from the voxels in the beam path. The sinogram, *i.e.* the chosen attribute as a function of *x* position and the projection angle, is used as the input for *tomopy*.

As examples, we have performed reconstructions [Fig. 6 ▸(*c*)] based on the intensity of the cellulose peak at 1.58 Å^−1^ (panel A) as well as the intensity at the *q* position (2.27 Å^−1^) devoid of any diffraction peaks (panel B), representing the abundance of crystalline cellulose and non-crystalline structural components, respectively. These two tomograms are very similar in appearance. We have therefore calculated the ratio between them, similar to how the cellulose crystallinity index is estimated in the literature. The resulting tomogram (panel C) shows that the crystallinity index is low in the pith and the bark, but high in the regions where vascular bundles are present. This is as expected and also observed in our measurements on physical sections of similar samples (data not shown). For comparison, we also reconstructed a tomogram (panel D) based on the logarithm of sample absorption derived from the beam intensity before and after the sample. In this case, high values appear in the bark, indicating higher material density, consistent with the higher overall scattering intensity observed in panels A and B.

## 4. Summary

We have described the instrumentation and software for scanning structural mapping at the LiX beamline. Further development of data processing software and its integration into data collection will continue, with the aim to lower the barrier for new users to start employing this technique. We are also exploring experiments that can exploit the low scattering background using in-vacuum scanning. In particular, we are testing measurements on individual plant cells. At the same time, we are also making an effort to communicate to the user community the availability of these measurements. Currently, we are taking advantage of the existing CBMS workbench (https://www.bnl.gov/cbmsworkbench/) designed to attract solution scattering and macromolecular crystallography users. As our user community grows, we expect to start a dedicated workbench for scanning structural mapping.

## Supplementary Material

Combined focal length of CRLs, as a function of X-ray energy and the number of lenses. DOI: 10.1107/S1600577521013266/ay5593sup1.pdf
