# Supplementary material for: Scanning structural mapping at the Life Science X-ray Scattering Beamline
Source: J Synchrotron Radiat. 2022 Jan 17;29(Pt 2):540–8. doi: 10.1107/S1600577521013266 (PMC8900859; doi:10.1107/S1600577521013266)
Supplement: Supplementary file 1 [file s-29-00540-sup1.pdf]

JOURNAL OF SYNCHROTRON RADIATION

Volume 29 (2022)

Supporting information for article:

Scanning structural mapping at the Life Science X-ray Scattering Beamline

Lin Yang, Jiliang Liu, Shirish Chodankar, Stephen Antonelli and Jonathan DiFabio

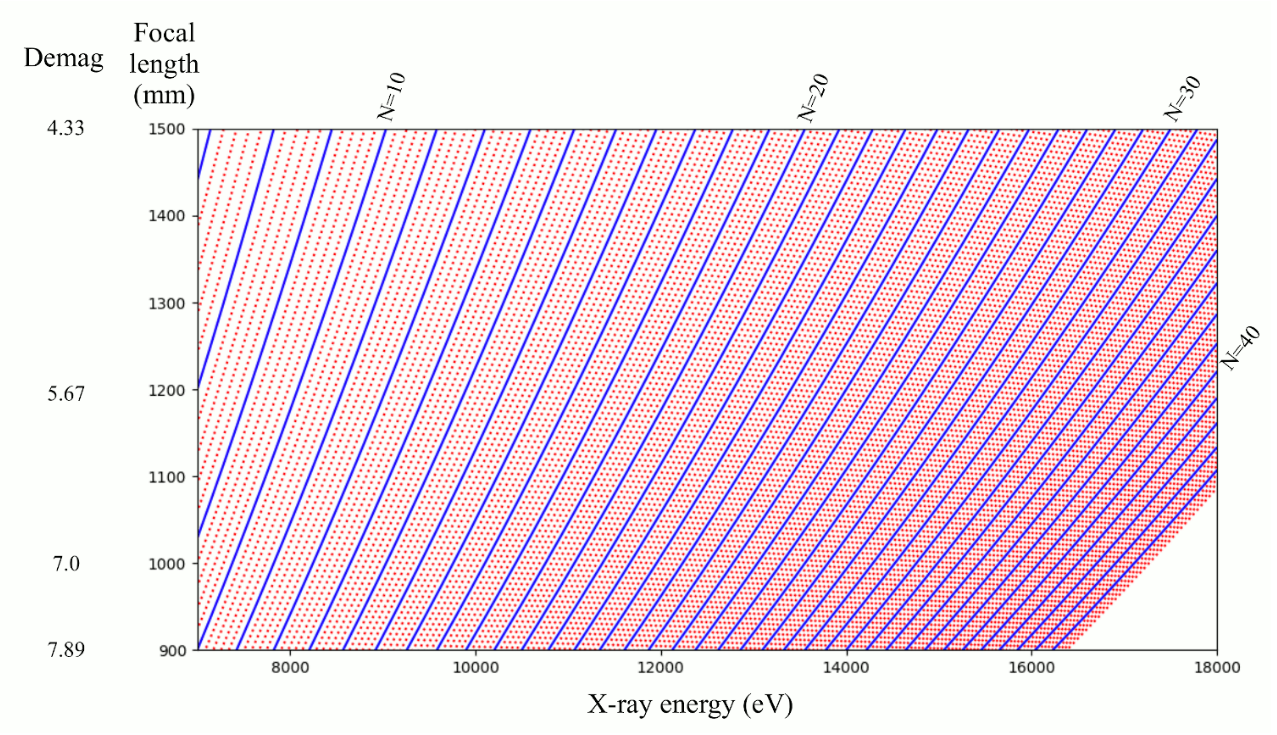

Figure S1. The combined focal length of CRLs, as a function of x-ray energy and the number of lenses. The corresponding demagnification ratio is also shown, given that the total distance between the secondary source and the sample is 8 m. The blue lines represent the focal length with 0.1 mm lenses only, with the number of lenses (N) noted for the configurations of N=10, 20, 30, and 40. The red dashed lines represent the results after adding the three 0.5mm lenses into the configuration. Since there are only 43 lenses of 0.1 mm radius, it is not possible to achieve focal lengths below 0.9 m at x-ray energies above 16.4 keV (lower right corner of the plot).
